# The relationship between patient's participation in shade selection and their satisfaction with their dental prostheses

**DOI:** 10.1002/cre2.508

**Published:** 2021-10-28

**Authors:** Faisal Alzeghaibi, Abdulaziz Jammah, Faisal Alghanim, Khalid Albawardi, Lubna Alkadi

**Affiliations:** ^1^ DMD, College of Dentistry King Saud bin Abdulaziz University for Health Sciences, National Guard Health Affairs Riyadh Saudi Arabia; ^2^ King Abdullah International Research Center Riyadh Saudi Arabia; ^3^ Restorative and Prosthetic Dental Sciences, College of Dentistry King Saud bin Abdulaziz University for Health Sciences, National Guard Health Affairs Riyadh Saudi Arabia

**Keywords:** color, patient participation, prosthesis, satisfaction

## Abstract

**Objectives:**

Multiple studies have measured the degree of patients' satisfaction with their dental prostheses. Some found that the preliminary cause for dissatisfaction was attributed to the shade used in the fabrication their prostheses. Therefore, the aim of this study is to evaluate the relationship between patients' participation in the shade selection procedure and their satisfaction with their prostheses.

**Material and Methods:**

In this cross‐sectional study, an online based questionnaire was distributed. Out of 475 participants, 374 who met inclusion criteria were enrolled in this study. A questionnaire consisting of 19 questions and two parts was utilized. Data were analyzed using IBM SPSS 25 with a level of significance (*p* value) ≤ 0.05.

**Results:**

The majority of participants were involved in the shade selection procedure (81%) and satisfied with the shade of their dental prostheses (83.7%). Satisfaction rate was higher in patients who participated in shade selection (87.8%). One in every three patients wanted a lighter shade than that they currently have. All results were statistically significant.

**Conclusion:**

Patients' participation in their prostheses' shade selection increased their satisfaction with them. It is crucial that patients receive professional advice during shade selection for them to make an informed decision regarding the shade of their prostheses.

## 1 INTRODUCTION

Nowadays, the general population is becoming increasingly aware of their overall appearance. Among the factors affecting patients' satisfaction and general acceptance of their overall appearance, dental esthetics is considered a cornerstone. Wagner et al, found that laypeople's assessment of their overall appearance was affected by their dental appearance, in which the shade of their teeth played a major role (Wagner et al., 1996). Samorodnitzky‐Naveh et al, surveyed a sample of 407 subjects and found that 37% were dissatisfied with the esthetic appearance of their teeth, particularly, the shade of their teeth (Samorodnitzky‐Naveh et al., 2007). Lombardi et al, reported that dental esthetics is governed by multiple factors, one of which is the shade, and is perceived differently between individuals (Lombardi, 1973). Samorodnitzky‐Naveh et al, in a cross‐sectional study compared 193 patients' self‐assessment of shade selection in relation to the clinician's assessment and found that only 18.7% of participants matched the clinician's selection of hue and chroma with women and non‐smokers achieving a closer match to their dentists' assessment compared to others. Only 3.6% were satisfied with their teeth, and 83.4% were interested in future bleaching (Samorodnitzky‐Naveh et al., 2010). Akarslan et al, investigated factors that influence patients' satisfaction with their current dental esthetics and previously received dental treatments on anterior teeth and inquired about the basic treatments that they plan to undergo to improve their dental appearance. They found that most of the patients assessed were dissatisfied with the shade (55.1%), followed by the dental appearance (42.7%) and crowding of anterior teeth (29.9%). Additionally, 23.3% habitually hide their teeth while smiling, 16.1% had non‐esthetic restorations and 11.9% thought perceived protrusion of their anterior teeth. In the same study, the most commonly performed treatment among participants was esthetic restorations and 49.0% of patients desired whitening of teeth. The study also reported that gender, age, and level of education influenced patients' satisfaction (Akarslan et al., 2009). Because shade selection is an influential factor on patients' satisfaction with their prostheses, this study aims to evaluate the relationship between patients' participation in the shade selection procedure and their satisfaction with their prostheses. Based on a literature search and to the best of our knowledge, this is the first study that tackles this issue.

## 2 MATERIAL AND METHODS

The aim of this cross‐sectional questionnaire‐based study is to evaluate the relationship between patients' participation in the shade selection procedure and their satisfaction with their prostheses. Ethical approval for the conduction of this study was obtained through Institutional Review Board from King Abdullah International Medical Research Center (RC19/294/R) was obtained in August 20, 2019. A questionnaire consisting of 19 questions and two parts was created initially in English and then translated to Arabic and was proofread by an English literature teacher who is fluent in both languages. Subsequently, the questionnaire was presented to content experts to ensure the questions were relevant to the aim of the study without repetition or overlap and achieved face‐validity. The questionnaire was then checked for reliability utilizing a pilot of 30 samples (15 participants), where each participant answered the questionnaire twice, then correlation coefficient test was applied, and all correlation coefficient *r* values were ≥ 0.70.

The first part of the questionnaire consisted of five questions related to demographics (gender, age group, educational level, nationality, and current employment status). The second part contained 14 questions inquiring about the presence and type of prostheses (veneers, anterior and posterior crowns or fixed dental prostheses, anterior or posterior implants, removable partial dentures, and complete dentures) as well as participants' satisfaction and involvement in the shade selection procedure. A 4‐point Likert Scale (agreement/disagreement) was utilized to measure satisfaction and degree of involvement in the shade selection procedure. A sample size of 374 was determined through a power calculation and collected through a convenience sampling technique. Each participant was assessed for compliance with study's inclusion criteria which were: age of 18 years or older, residence in Riyadh, Saudi Arabia, and possession of one or more of the following types of prostheses: veneers, anterior and posterior crowns or fixed dental prostheses, anterior or posterior implants, removable partial dentures, or complete dentures. The questionnaire was created and distributed through online based google forms with Informed Consent form attached. Of the 475 responses that were received, 101 responses were excluded as they failed to meet the inclusion criteria. All collected data were entered and analyzed using IBM SPSS version 25. Categorical variables were summarized and reported in terms of frequency distribution, and all variables were statistically compared using Chi‐square test utilizing a level of significance (*p‐*value) of 0.05 or less.

## 3 RESULTS

**Figure 1:**
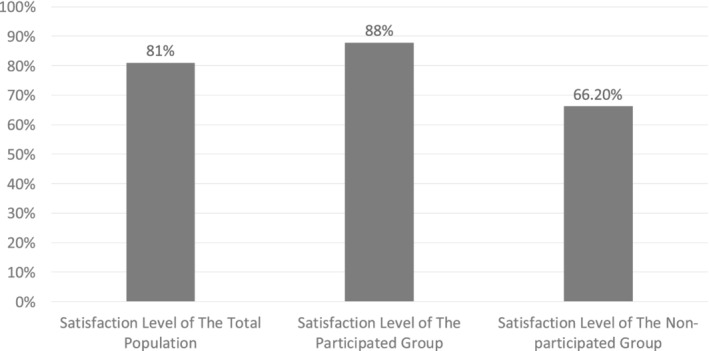
Patients satisfaction levels towards their prosthesis shade in three groups

Out of the 374 participants, 81% were involved in the shade selection for their dental prostheses. Out of those involved, 87.8% were satisfied with their prosthesis shade. The percentage of satisfaction of the total population was 83.7%. On the other hand, the satisfaction level dropped to 66.2% in the group of patients that were not involved in shade selection (*p‐*value = 0.001) (Figure 1).

Among 374 questionnaires distributed, 70.3% were females. The frequency distribution of participants' demographics (age groups, educational level, and employment status) is given in Table 1. The most dissatisfied age group with their prosthesis were those between 18 and 29 years old (34) (*p‐*value = 0.003). Likewise, Students were among the most dissatisfied (33.3%) (*p*‐value = 0.031). Table 2 demonstrates the frequencies of each treatment modality patients had received.

**Table 1:** Frequency and distribution of demographics

	Frequency (*n* = 374)	Percentage (100)
Age group		
18–29	50	13.4
30–39	79	21.1
40–49	119	31.8
50 and above	126	33.7
Educational level		
Elementary	7	1.9
Secondary	14	3.7
High school	54	14.4
Diploma	47	12.6
Bachelor	226	60.4
Master's degree	18	4.8
PhD	8	2.1
Employment status		
Student	21	5.6
Retired	79	21.1
Not employed	96	25.7
Employed	178	47.6

**Table 2:** Frequency and percentage of each treatment modality among participants

	Frequency (*n =* 527)	Percentage (100)
Veneers	39	7.4
Anterior crowns	123	25.04
Posterior crowns	212	40.22
Anterior implant	37	7.02
Posterior implant	90	17.07
RPD	18	3.42
Complete denture	8	1.5

Furthermore, 76.2% of the participants confirmed that their shade selection decision was influenced by their dentist. Although a high percentage of the participants (83.7%) were satisfied with the shade of their dental prosthesis, around one third (35.8%) would prefer the prosthesis to be lighter, while only (16%) would prefer it to be darker. Almost half of the participants thought that their eyes and skin color affect the prosthesis shade selection (52.9%), and 74.3% agreed that the shade of the prosthesis will affect their overall appearance. Interestingly, a low percentage of the participants agreed that celebrities indirectly influence their shade selection (23%).

**Figure 2:**
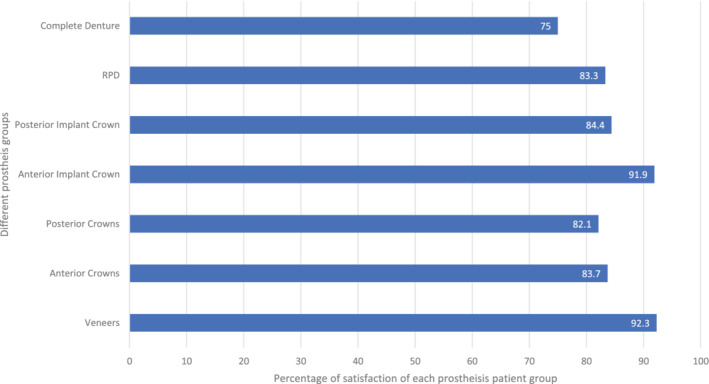
The level of satisfaction for each group of prosthetic treatment

When the association between the level of satisfaction to the type of prostheses was statistically analyzed, all were statistically insignificant with a *p‐*value higher than 0.05. However, the highest satisfaction rate with shade was noted in patients who received porcelain laminate veneers (92.3%), followed by patients with anterior implant crowns (91.9%) (Figure 2). Likewise, the setting in which the patient received their dental prosthesis (whether in a private practice, governmental hospital, or a university hospital) was not statistically significant when compared with shade satisfaction level. Yet, patients that received their prosthesis in university dental hospitals were the most satisfied group.

## 4 DISCUSSION

This study was conducted to evaluate the relationship between patients' participation in shade selection and their satisfaction with their dental prostheses. As previously stated, the color of the dentition or the dental prostheses has a major impact on the patient's level of satisfaction (Samorodnitzky‐Naveh et al., 2007; Wagner et al., 1996).

The current study confirms that patients' involvement in the process of shade selection correlates positively with their satisfaction with their prostheses. In addition, the majority of participants stated that the shade of their prostheses affected their overall well‐being and appearance. Moreover, one‐third of the participants, although satisfied, desired a lighter shade for their prostheses. Although this desire could be attributed to the influence of social media and celebrities, only a small number of participants confirmed the presence of such an influence.

Furthermore, although statistically insignificant, participants who have porcelain laminate veneers were the most satisfied with their prostheses shade. Generally, the results failed to demonstrate statistical significance between satisfaction rate and possession of different prostheses. This may be related to the small number acquired for each subgroup after dividing the sample.

In the current study, the majority of participants agreed that teeth appearance had a great effect on their overall appearance. This finding is in agreement with Wagner et al. who also found that the majority of patients and dentists had conflicting opinion regarding shade selection. This may explain why patients who actively participated in the shade selection procedure demonstrated higher satisfaction rates (Wagner et al., 1996).

In this article, the data collection approach was similar to previous studies where a self‐reporting questionnaire was filled by the patients (Lombardi, 1973; Samorodnitzky‐Naveh et al., 2007; Samorodnitzky‐Naveh et al., 2010; Vallittu et al., 1996; Wagner et al., 1996). A Likert scale was utilized in the current study similar to the studies done by Wagner et al and Vallittu et al. (Vallittu et al., 1996; Wagner et al., 1996). However, unlike Vallittu et al, the current study utilized a 4‐point scale (strongly agree, agree, disagree, and strongly disagree) for each statement rather than the more commonly used 5‐point scale. This was made following the analysis of data collected from a pilot questionnaire where results showed a high tendency for participants to choose the middle (neutral) option. To eliminate this central tendency, the middle option was eliminated. Although Wagner et al, used a 4‐point scale as we did they were giving the patients different statement that they should show there opinion on the level of importance. On the other hand, some other studies have used direct questions with yes and no options (Akarslan et al., 2009; Samorodnitzky‐Naveh et al., 2007; Samorodnitzky‐Naveh et al., 2010).

Based on the results of this study, in order to achieve the highest satisfaction rate possible, restorative dentists are encouraged to give their patients autonomy in selecting the shade of their prostheses, while providing them with sufficient professional guidance to assist them in making an informed decision.

## 5 CONCLUSION

After surveying patients with dental prostheses of all types, who were treated in different clinical settings, the majority of patients have participated in the shade selection process. Patients' participation played a critical role in increasing their overall satisfaction with the treatment rendered.

## CONFLICT OF INTEREST

The authors declare no conflicts of interest.

## AUTHOR CONTRIBUTIONS

All authors have contributed in the study design and concept. As well as data collection. Data analysis was done by Abdelmoneim M. Eldali. The manuscript first draft was written by Faisal Alzeghaibi, and has been corrected and integrated by Lubna Alkadi. All authors read and approved the final manuscript.
